# Variational Sparse Bayesian Learning for Estimation of Gaussian Mixture Distributed Wireless Channels

**DOI:** 10.3390/e23101268

**Published:** 2021-09-28

**Authors:** Lingjin Kong, Xiaoying Zhang, Haitao Zhao, Jibo Wei

**Affiliations:** School of Electronic Science and Engineering, National University of Defense Technology, Changsha 410073, China; konglingjin19@nudt.edu.cn (L.K.); haitaozhao@nudt.edu.cn (H.Z.); wjbhw@nudt.edu.cn (J.W.)

**Keywords:** channel measurement, Gaussian mixture model, variational Bayesian, sparse channel, channel estimation

## Abstract

In this paper, variational sparse Bayesian learning is utilized to estimate the multipath parameters for wireless channels. Due to its flexibility to fit any probability density function (PDF), the Gaussian mixture model (GMM) is introduced to represent the complicated fading phenomena in various communication scenarios. First, the expectation-maximization (EM) algorithm is applied to the parameter initialization. Then, the variational update scheme is proposed and implemented for the channel parameters’ posterior PDF approximation. Finally, in order to prevent the derived channel model from overfitting, an effective pruning criterion is designed to eliminate the virtual multipath components. The numerical results show that the proposed method outperforms the variational Bayesian scheme with Gaussian prior in terms of root mean squared error (RMSE) and selection accuracy of model order.

## 1. Introduction

Channel modeling plays a pivotal role in the design and evaluation of wireless communication networks. In the fifth-generation (5G) mobile communication systems, the diversity of the communication scenarios leads to the urgent need for advanced channel modeling techniques with higher accuracy and lower implementation complexity. In the existing channel measurement campaigns, the channel sounding technique based on pseudo-random (PN) sequences has widely been adopted due to its higher processing gain and delay resolution. As for the estimation of the multipath characteristics such as power delay profile, Doppler power spectrum, and frequency response from the obtained channel responses, super-resolution algorithms such as space-alternating generalized expectation-maximization (SAGE) [1] and subtractive deconvolution technique (CLEAN algorithm) [2] are widely applied. However, since the SAGE and CLEAN algorithms are based on the maximum likelihood criterion, the selection of model order, i.e., the number of multipath, is a nontrivial task. The estimated order of the channel model tends to be overestimated because of the contamination of noise. The unnecessary “virtual paths” will not only affect the accuracy of the channel coefficients estimation but also increase the computational cost of the algorithm. To solve this problem, different techniques such as negative log-evidence (NLE) [3] and Bayesian information criterion (BIC) [4] have been proposed to estimate the model order. However, the estimation performance of these model estimation schemes is far from ideal, especially when considering the low signal-to-noise ratio scenarios [5]. Since considerable computation is needed to find the optimal model order value, the existing techniques also suffer from prohibitive complexity.

Due to the fact that the multipath components (MPC) are sparse in channel impulse responses (CIR) [6], the joint estimation problem of channel parameters and channel model order can be addressed by sparse Bayesian learning (SBL). SBL is a typical machine learning algorithm that was first proposed by Tipping in 2001 [7] and has been developed into an important branch of compressed sensing reconstruction algorithms. The basic idea of SBL is to introduce the sparsity parameter to establish a hierarchical Bayesian model. By defining the priors on the sparse vector, SBL can automatically determine the position and amount of the non-zero elements in the reconstructed signal without pre-specifying the sparsity number. SBL is a promising method for the joint estimation of channel model order and the channel response coefficients. However, even after the hierarchical Bayesian model is established, the posterior PDF cannot be directly derived due to an intractable integral of the joint probability density. Thus, the variational inference is introduced to obtain an approximation of the parameter posterior in the SBL framework. This method, also known as variational sparse Bayesian learning (VSBL) [8], has been widely applied in online spectrum estimation [9], orthogonal time-frequency space (OTFS) detector [10], multi-user joint decoding [11], joint signal detection [12], and high-resolution radar imaging [13], etc. For channel parameter estimation, the idea of complete hidden data is introduced to speed up the convergence in [5]. The simulation result shows that the VSBL scheme outperforms the BIC criterion in both root mean squared error (RMSE) and model order selection. In [14], the delay of MPCs is modeled as the Poisson distribution, which incorporates the Fast VSBL based channel estimation as a priori information and significantly improves the bit error ratio (BER) performance.

Several channel measurement campaigns conducted in high-speed railways [15], airport grounds [16,17], and inland rivers [18] have shown that Weibull and Nakagami-m distributions are more suitable for modeling channel characteristics compared with Rayleigh and Rice distribution. This phenomenon originates from the uneven distribution of scatterers in the mobile communication environment. However, in most VSBL models, the channel taps were assumed to be Gaussian distributed [19,20], which is not appropriate for parameter learning. In this paper, the Gaussian mixture model (GMM) is adopted to describe the statistical characteristics of the MPCs. A GMM is linearly composed of K Gaussian PDFs with corresponding means and covariances. By using sufficient Gaussian components, the GMM can be flexible to approximate any given PDF [21], which is suitable for establishing various fading channels models.

The contributions of this paper are summarized as follows:

- The Gaussian mixture model as a powerful method for sparse parameter learning for wireless channels is introduced to the estimation problem of wireless channel parameters under the VSBL framework. The flexibility of GMM is capable of describing the statistical characteristics of both theoretical general channels and complex real-world channels.
- A new variational Bayesian inference scheme for the Gaussian mixture model (VB-GMM) is developed based on multiple channel observations. The corresponding graphical model is given, and the closed-form updates of the model variables are derived. By setting a pruning criterion on the sparsity priors, the joint estimation of channel parameters and model order is achieved with low complexity.
- The simulation results demonstrate that the performance of VB-GMM is superior to the existing algorithms in terms of the estimation error, the convergence rate, and the model order selection accuracy in most non-Gaussian channels.

Throughout the paper, we use the following notations. ${\left(\cdot \right)}^{H}$ and ${\left(\cdot \right)}^{-1}$ denote the Hermitian transpose and the inverse of matrices, respectively. The expression $CN\left(\mu ,\Sigma \right)$ represents the multivariate complex Gaussian distribution with mean vector μ and covariance matrix Σ. $Ga\left(\alpha |a,b\right)$ denotes the Gamma distribution $p\left(\alpha \right)=\frac{b^{a}}{\Gamma \left(\alpha \right)}\cdot \alpha^{a-1}e^{-b\alpha },$ α>0 in which a, b and $\Gamma \left(\cdot \right)$ represent shape parameter, scale parameter and Gamma function, respectively. We use calligraphic uppercase letters to represent sets, e.g., I denotes the index set of MPCs and $\bar{I(l)}$ is the complement set of l∈I. The remainder of the paper is organized as follows. In Section 2, the signal model is introduced, and the graphical model of VB-GMM is presented. In Section 3, the derivations of the VB-GMM estimators are given followed by the initialization algorithm and the pruning criterion. Finally, in Section 4, the numerical results are presented to demonstrate the effectiveness of the proposed scheme.

## 2. System Model

### 2.1. Signal Model

Consider a single-input-single-output (SISO) wireless channel measurement system, the sounding signal is denoted as $s\left(t\right)=\sum_{i=0}^{N_{C}-1}p_{i}b(t-iT_{c})$, in which the pseudo-random sequence $\left\{p_{i}\right\},i=0,\ldots ,N_{C}-1$ has $N_{C}$ chips and the shaped pulse b(t) has the duration $T_{c}$. Assume that $N_{S}$ copies of $s\left(t\right)$ are transmitted periodically to obtain the time-varying channel responses. After passing through the multipath channel, the signal at the receiver is made of L duplications of $s\left(t\right)$ with additive Gaussian white noise $\eta \left(t\right)$, and the received signal corresponding to the *n*-th $s\left(t\right)$ can be expressed as

(1) $$y_{n}\left(t\right)\;=\sum_{l=1}^{L}w_{ln}s\left(t,\theta_{\ln}\right)\;+\;\eta \left(t\right),n=1,\ldots ,N_{S}$$

where $w_{ln}$ is the complex coefficient of the l-th multipath and $\theta_{ln}$ denotes the dispersion parameter vector including multipath delay, DoA, DoD, etc. We define $y_{n}={\left[y_{n}\left(0\right),y_{n}\left(T_{s}\right),\ldots ,y_{n}\left(\left(D-1\right)T_{s}\right)\right]}^{T}$ as the discrete samples of the *n*-th received signal in which $T_{s}$ is the sampling interval at the receiver and D represents the number of discrete samples, i.e., $DT_{s}=N_{c}T_{c}$. The discrete vector $y_{n}$ is considered as an observation of the measured channel. In our proposed scheme, an Bayesian probabilistic model is established to infer the posterior probabilities of the channel parameters $\left\{w_{ln},\theta_{ln},L\right\}$ based on multiple observations.

Since the proposed algorithm is developed based on time-varying channels, it is crucial to determine the wide-sense stationary (WSS) region in which the statistical characteristics of channel parameters are assumed invariant. For vehicle-to-vehicle channel, the spatial distance of 20~40 times the wavelength is considered to be WSS [22]. For convenience, we denote the multiple observation matrix as $y={\left[y_{1},y_{2},\ldots ,y_{N}\right]}_{D\times N}$, where N represents the number of channel responses obtained in a WSS region. If the spatial distance of the WSS is chosen properly according to the criterion, the dispersion parameter vector is assumed to be constant for the observations, i.e., $\theta_{l1}=\ldots =\theta_{lN}=\theta_{l},n=1,\ldots ,N$. Thus Equation (1) can be rewritten in a discrete form as:

(2) $$y_{n}=\sum_{l=1}^{L}w_{ln}s(\theta_{l})+\eta$$

in which $\eta \text{\textasciitilde{}}CN\left(0,\Sigma \right)$ is the Gaussian white noise vector.

In the SAGE algorithm, the complete parameter set Ω is divided into several subsets $\Omega_{\mathrm{sub}}$ so that the joint estimation for Ω is decomposed into optimizations for $\Omega_{\mathrm{sub}}$ in a sequential manner. Let $x_{\mathrm{sub}}$ denote the admissible hidden data with respect to $\Omega_{\mathrm{sub}}$, when the equation $p\left(y\left|x_{\mathrm{sub}},\Omega \right.\right)=p\left(y\left|x_{\mathrm{sub}},\Omega_{\bar{\mathrm{sub}}}\right.\right)p\left(x_{\mathrm{sub}}\left|\Omega \right.\right)$ is satisfied, the likelihood corresponding to the estimation monotonically increases and may converge to the local maximum [23]. In this paper, the concept of admissible hidden data $x_{l}$ is also adopted. Consider an MPC l we have

(3) $$x_{l}=w_{l}s(\theta_{l})+\eta_{l}$$

in which $\eta_{l}$ represents the noise that the *l*-th MPC contribute to the total noise η with $E\left\{\eta_{l}\eta_{l}^{H}\right\}=\Sigma_{l}=\beta \Sigma$, 0≤β≤1, and the likelihood $p(x_{l}|\theta_{l},w_{l})=CN(x_{l};w_{l}s(\theta_{l}),\Sigma_{l})$. Then the covariance of the noise that is not related to the l-th component is $\Delta_{l}=\left(1-\beta \right)\Sigma$, which can also affect the estimation of $x_{l}$ when observations are obtained and fixed. The noise covariance has a significant impact on the estimation results of channel parameters. Since the length of the channel sounding frame is generally much longer than the channel delay spread, most of the channel response samples are considered to be measurement noise. In this paper, the noise covariance is computed by the tail of CIRs.

### 2.2. The GMM Model of Channel Coefficients

Denote the N channel coefficients of the l-th multipath as $w_{l}={\left[w_{l1},w_{l2},\ldots ,w_{lN}\right]}_{1\times N}$. Assume that $w_{l}$ obeys the Gaussian mixture distribution with K components:

(4) $$p\left(w_{l}\left|\pi_{l},\mu_{l},\Lambda_{l}\right.\right)=\sum_{k=1}^{K}\pi_{lk}CN\left(w_{l}|\mu_{lk},\Lambda_{lk}\right)$$

where $\pi_{l}=[\pi_{l1},\pi_{l2},\ldots ,\pi_{lK}],\;\sum_{k=1}^{K}\pi_{lk}=1$ is defined as mixing coefficients. $\mu_{lk}$ and $\Lambda_{lk}$ represent the complex mean and covariance matrix of the k-th Gaussian distribution, respectively. For $w_{l}$, each sample can only be derived from one component of the Gaussian mixture distribution. Therefore, the discrete hidden variable $c_{l}$ is introduced. $c_{l}$ is a 0–1 matrix in which $c_{lnk}=1$ means the channel coefficient $w_{ln}$ corresponds to the k-th Gaussian component. Given the mixing coefficients, the probability of hidden variables takes a product form:

(5) $$p\left(c_{l}\left|\pi_{l}\right.\right)=\prod_{n=1}^{N}\prod_{k=1}^{K}\pi_{lk}^{c_{lnk}}$$

For the variable $\pi_{l}$ a Dirichlet distribution prior is applied:

(6) $$p(\pi_{l}|\gamma_{l})=Dir(\pi_{l}|\gamma_{l})=C(\gamma_{l})\prod_{k=1}^{K}\pi_{lk}^{\gamma_{lk}-1}$$

where $C(\gamma_{l})=\frac{\Gamma \left(\gamma_{l0}\right)}{\prod_{k=1}^{K}\Gamma \left(\gamma_{lk}\right)},\;\;\gamma_{l0}=\sum_{k=1}^{K}\gamma_{lk}$, and $\gamma_{l}=\left[\gamma_{l1},\ldots ,\gamma_{lK}\right]$ is assumed non-informative, i.e., $\gamma_{l1}=\gamma_{l2}=\cdots =\gamma_{lK}$. The Dirichlet distribution is introduced here as the conjugate prior of the polynomial likelihood function $p\left(c_{l}\left|\pi_{l}\right.\right)$, which means the posterior of $c_{l}$ has the same form of Dirichlet distribution. In variational inference, the application of conjugate priors makes it convenient to merely update the parameters of the density functions with their form unchanged.

**Figure 1:**
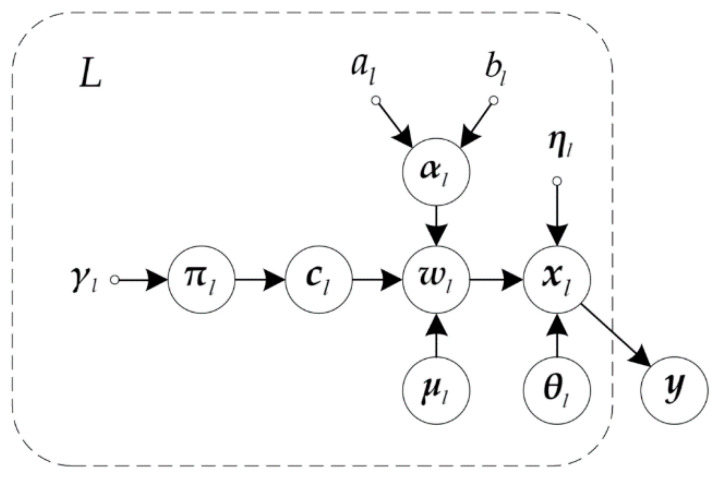
Graphical Model for VB-GMM algorithm.

The graphical model for the variational Bayesian-based GMM is presented in Figure 1. Denote the parameter set as $\Omega =\left\{\theta ,w,\mu ,c,\alpha ,\pi \right\}$ the joint probability density can be rewritten according to the structure of the graph:

(7) $$p\left(y,\Omega \right)=p\left(y\left|w,\theta ,\eta \right.\right)p\left(w\left|\alpha \right.,c,\mu \right)p\left(c,\pi \right)p\left(\alpha \right)p\left(\mu \right)$$

in which

(8) $$p\left(y\left|w,\theta ,\eta \right.\right)=\prod_{n=1}^{N}CN(y_{n};\sum_{l=1}^{L}w_{ln}s(\theta_{l}),\eta )$$

(9) $$p\left(w\left|\alpha \right.,c,\mu \right)=\prod_{l=1}^{L}p\left(w_{l}\left|\alpha_{l},c_{l},\mu_{l}\right.\right)$$

The sparsity prior of l-th path $p\left(w_{l}\left|\alpha_{l},c_{l},\mu_{l}\right.\right)$ is a key to restrain the gain of MPCs and implement model order selection. The sparsity prior is usually chosen as Laplace [24] or Gaussian distributed [7]. The Laplace prior leads to a $l_{1}$-type sparsity and Gaussian prior a $l_{2}$-type sparsity [5]. In the proposed scheme each component of the GMM is specified with an independent Gaussian sparsity prior, i.e., $p\left(w_{ln}\left|\alpha_{lk};c_{lnk}=1\right.\right)=CN(\mu_{lk},\alpha_{lk}^{-1})$. Thus consider all the observations for the l-th path, we define

(10) $$p\left(w_{l}\left|\alpha_{l},c_{l},\mu_{l}\right.\right)=\prod_{n=1}^{N}\prod_{k=1}^{K}{\left\{\frac{1}{\sqrt{2\pi \alpha_{lk}^{-1}}}\exp\left(-\frac{{\left(w_{ln}-\mu_{lk}\right)}^{2}}{2\alpha_{lk}^{-1}}\right)\right\}}^{c_{lnk}}$$

The nonnegative sparsity parameter $\alpha_{lk}$ is inversely proportional to the width of the Gaussian function. Consider a situation that $\alpha_{lk}\to \infty$, the Gaussian function will be concentrated at the mean value $\mu_{lk}$, which renders the MAP estimations of channel coefficients to be constant. Due to the time-varying nature of wireless channels, the channel coefficients cannot be a constant value over time, unless they are concentrated at the value of zero, i.e., the l-th MPC is considered to be the measurement noise and should be removed. By setting a large threshold for $\alpha_{l}$, the irrelevant MPCs are pruned and the sparsity is obtained. The prior of the sparsity parameter $p\left(\alpha_{lk}\right)$ is set to be a Gamma distribution $p\left(\alpha_{lk}\right)=Ga\left(\alpha_{lk}|a_{lk},b_{lk}\right)$ with a non-informative hyperprior for all branches, i.e., $a_{lk}=b_{lk}=10^{-7}$, k=1,2,…,K. Such choice is proper when no prior information is available, and it has been widely applied in various Bayesian hierarchical models, e.g., [5,7,8].

## 3. GMM-Based Variational Bayesian Learning

In this section, the VSBL is applied to the estimation problem of the Gaussian mixture channel model. First, the basic principle of variational Bayesian inference is introduced. Then the update expression of each variable in the graphical model is derived. Lastly, the appropriate initialization algorithm and corresponding parameter settings are given.

### 3.1. Variational Bayesian Inference

Consider a probabilistic model defined by observed variables O, hidden variable sets H, and the corresponding joint probability density $p\left(H,O\right)$. Our target is to obtain the posterior $p\left(H\left|O\right.\right)=p\left(H,O\right)/p\left(O\right)$. However, the computation of the marginal likelihood function $p\left(O\right)$ is intractable since it involves a multi-dimensional and complicated integral $\int p\left(H,O\right)dH$. Therefore, a PDF with a simpler structure is introduced to approximate the posterior distribution, i.e.,

(11) $$q\left(H\right)\approx p\left(H\left|O\right.\right)$$

where $q\left(H\right)$ is the variational posterior distribution (VPD). In the variational Bayesian framework, the Kullback-Leibler (KL) divergence is defined as

(12) $$\mathrm{KL}(q\left\|p\right.)=\int q\left(H\right)\ln\left\{\frac{q\left(H\right)}{p\left(H\left|O\right.\right)}\right\}dH$$

To obtain a deterministic approximation of the posterior, (12) should be minimized, and the optimal VPD $q^{*}\left(H\right)$ is achieved only if $\mathrm{KL}(q\left\|p\right.)=0$. Using Bayes theorem to replace $p\left(H\left|O\right.\right)$, it makes the derivation of VPD a tractable optimization problem:


(13) $$q^{*}\left(H\right)=\arg\underset{q\left(H\right)}{\max}\int q\left(H\right)\ln\left\{\frac{p\left(H,O\right)}{q\left(H\right)}\right\}dH$$

For simplicity, we adopt the mean-field theory and assume the joint PDF factorizes as

(14) $$q\left(H\right)=\prod_{s=1}^{S}q_{s}\left(H_{s}\right)$$

For most of the variables including $\left\{w,\alpha ,c,\pi ,x\right\}$, (13) is optimized over the exponential family of distributions. In this case, the KL divergence vanishes, and the optimal solution of (13) is assumed to be achievable. For μ and θ, the optimization on $q_{s}\left(H_{s}\right)$ is restricted on Dirac measures to obtain point estimates. By optimizing (13), the solution that minimizes the KL divergence is:


(15) $$q_{s}^{*}\left(H_{s}\right)\propto \exp\left(E_{q\left(MB\left\{H_{s}\right\}\right)}\ln\left\{p\left(H,O\right)\right\}\right)$$

In (15), $E_{q\left(x\right)}\left(\cdot \right)$ denotes the expectation with respect to the distribution $q\left(x\right)$,$MB\left\{H_{s}\right\}$ represents the Markov blanket [25] of the variable $H_{s}$. In the Bayesian network, the Markov Blanket of a node is the set of the nodes comprised of the node’s parents, its children, and its co-parents. I.e., $MB\left\{H_{s}\right\}$ is the minimal set around $H_{s}$ which makes $H_{s}$ independent of the rest of the variables.

### 3.2. Update of the Estimation Expressions

As discussed in Section 2, a hierarchical Bayesian model is set up to find sparse MPCs and infer the parameter sets $\Omega =\left\{\theta ,w,\mu ,c\right\}$ from the measurements y. A detailed derivation of the VPDs for each factor is given below. The VPDs can be updated in any order during the iteration process.

1. *Estimation of*$q(x_{l})$: From the graphical model in Figure 1 and (15), the VPD of $x_{l}$ is expressed as $q\left(x_{l}\right)\propto \exp\left(E_{q\left(w_{I}\right)q\left(\theta_{I}\right)}\left(\ln\left\{p(x_{l}|y,\theta_{I},w_{I})\right\}\right)\right)$, in which
  (16) $$p(x_{l}|y,\theta_{I},w_{I})=p(x_{l}|\theta_{l},w_{l})p(x_{l}|y,\theta_{\bar{I\left(l\right)}},w_{\bar{I\left(l\right)}})$$

In (16), I is the set of all MPCs. Note that scattering parameters $\theta_{l}$ should be determined for a specific MPC, thus, we define $q(\theta_{l})$ to be a point estimation, i.e., $q(\theta_{l})=\delta (\theta_{l}-\;{\hat{\theta }}_{l})$, where $\;{\hat{\theta }}_{l}$ denotes the estimated value for $\theta_{l}$ in a former update iteration. Based on the Gaussian noise assumption in Section 2.1, we have

(17) $$p(x_{l}|y,\theta_{\bar{I(l)}},w_{\bar{I(l)}})=CN(x_{l}|y-\sum_{p\in \bar{I(l)}}w_{p}s(\theta_{p}),\Delta_{l})$$

By substituting $p(x_{l}|\theta_{l},w_{l})$ and (17) into (16), the admissible hidden data of the n-th observation obeys a Gaussian distribution with the mean and covariance matrix as follows:

(18) $${\hat{x}}_{ln}^{\prime }=\left(1-\beta \right){\hat{w}}_{ln}s({\hat{\theta }}_{l})+\beta \left(y_{n}-\sum_{p\in \bar{I(l)}}{\hat{w}}_{pn}s({\hat{\theta }}_{p})\right)$$

(19) $${\hat{S}}_{l}^{\prime }={\left(\Delta_{l}^{-1}+\Sigma_{l}^{-1}\right)}^{-1}$$

2. *Estimation of*$q(c_{l})$: Evaluating (15) with (5) and (10), the VPD of $c_{l}$ takes the form
  (20) $$q(c_{l})\propto \prod_{n=1}^{N}\prod_{k=1}^{K}r_{lnk}^{c_{lnk}}$$
  in which
  (21) $$r_{lnk}=\exp\left(\psi ({\hat{\gamma }}_{lk})-\psi (\sum_{k=1}^{K}{\hat{\gamma }}_{lk})+\frac{1}{2}\left[\psi ({\hat{a}}_{lk})-\ln({\hat{b}}_{lk})\right]-\frac{{\hat{\alpha }}_{lk}}{2}\left|{\hat{w}}_{ln}^{2}+{\hat{\Phi }}_{ln}^{\prime }-2{\hat{\mu }}_{lk}{\hat{w}}_{ln}+{\hat{\mu }}_{lk}^{2}\right|\right)$$
  and $\psi \left(\cdot \right)$ represents the di-gamma function. Since $c_{l}$ is a 0–1 matrix with a discrete VPD, the posterior expectation of $c_{lnk}$ is $E_{q(c_{l})}\left\{c_{lnk}\right\}=q(c_{lnk}=0)\times 0+q(c_{lnk}=1)\times 1=r_{lnk}$.

3. *Estimation of*$q(w_{l})$: From the graph the Markov blanket of $w_{l}$ is $\left\{x_{l},\alpha_{l},\theta_{l},c_{l},\mu_{l}\right\}$, thus
  (22) $$q(w_{l})\propto \exp\left(E_{q\left(ℳℬ\left\{w_{l}\right\}\right)}\left(\ln\left\{p(x_{l}|\theta_{l},w_{l})p(w_{l}|\alpha_{l},c_{l},\mu_{l})\right\}\right)\right)$$

By integrating the factors in $MB\left\{w_{l}\right\}$, the VPD can be written as $q(w_{l})=\prod_{n=1}^{N}q(w_{ln})=\prod_{n=1}^{N}CN\left({\hat{w}}_{ln}^{\prime },\;{\hat{\Phi }}_{ln}^{\prime }\right)$ with

(23) $${\hat{w}}_{ln}^{\prime }={\hat{\Phi }}_{ln}^{\prime }\left(s{\left({\hat{\theta }}_{l}\right)}^{H}\Sigma_{l}^{-1}\;{\hat{x}}_{ln}+\sum_{k=1}^{K}{\hat{\mu }}_{lk}{\hat{r}}_{lnk}{\hat{\alpha }}_{lk}\right)$$

(24) $${\hat{\Phi }}_{ln}^{\prime }={\left(\sum_{k=1}^{K}{\hat{r}}_{lnk}{\hat{\alpha }}_{lk}+s{\left({\hat{\theta }}_{l}\right)}^{H}\Sigma_{l}^{-1}s({\hat{\theta }}_{l})\right)}^{-1}$$

4. *Estimation of*$q(\theta_{l})$: Similarly, evaluating (15) leads to $\tilde{p}(\theta_{l})\propto$ $p(\theta_{l})\exp\left(E_{q(x_{l})}{}_{q(w_{l})}\left(\ln\left\{p(x_{l}|w_{l},\theta_{l})\right\}\right)\right)$. As mentioned above, the proxy PDF is defined as $q(\theta_{l})=\delta (\theta_{l}-\;{\hat{\theta }}_{l})$ in order to obtain a point estimation. Therefore, we have
  (25) $${\hat{\theta }}_{l}^{\prime }=\arg\underset{\theta_{l}}{\max}\left(\ln p(\theta_{l})+\sum_{n=1}^{N}\left|{\hat{w}}_{ln}s({\hat{\theta }}_{l})\Sigma^{-1}{\hat{x}}_{ln}^{H}\right|\right)$$

5. *Estimation of*$q(\alpha_{l})$: From the graphic model the Markov blanket of $\alpha_{l}$ is $MB\left\{\alpha_{l}\right\}=\left\{w_{l},c_{l},\mu_{l}\right\}$ and $q(\alpha_{l})\propto p(\alpha_{l})\exp\left(E_{q(w_{l},c_{l},\mu_{l})}\left(\ln\left\{p(w_{l}|\alpha_{l},c_{l},\mu_{l})\right\}\right)\right)$. Due to the gamma hyperprior $p(\alpha_{l})=Ga(\alpha_{lk}|a_{lk},b_{lk})$ and the nature of conjugate prior, the VPD also satisfies the Gamma distribution, i.e., $q(\alpha_{lk})\propto Ga(\alpha_{lk}|{\hat{a}}_{lk}^{\prime },{\hat{b}}_{lk}^{\prime })$ with
  (26) $${\hat{a}}_{lk}^{\prime }=a_{lk}+\frac{1}{2}\sum_{n=1}^{N}{\hat{r}}_{lnk}$$
  (27) $${\hat{b}}_{lk}^{\prime }=b_{lk}+\sum_{n=1}^{N}{\hat{r}}_{lnk}\frac{\left|{\hat{w}}_{ln}^{2}+{\hat{\Phi }}_{ln}-2{\hat{\mu }}_{lk}{\hat{w}}_{ln}+{\hat{\mu }}_{lk}^{2}\right|}{2}$$

6. *Estimation of*$q(\pi_{l})$: The only variable related to $\pi_{l}$ is $c_{l}$, thus, $q(\pi_{l})\propto p(\pi_{l})\exp\left(E_{q(c_{l})}\left\{\ln p(c_{l}|\pi_{l})\right\}\right)$. By substituting (5) and (6) into (15) we obtain
  (28) $$q(\pi_{l})\;\propto \prod_{k=1}^{K}\pi_{k}^{\gamma_{lk}+\sum_{n=1}^{N}r_{lnk}-1}$$
  which indicates that the VPD also satisfies the Dirichlet distribution. The parameter of the VPD can be updated by
  (29) $${\hat{\gamma }}_{lk}^{\prime }=\gamma_{lk}+\sum_{n=1}^{N}r_{lnk}$$

7. *Estimation of*$q(\mu_{l})$: The Markov blanket of $\mu_{l}$ is $MB\left\{\mu_{l}\right\}=\left\{w_{l},c_{l},\alpha_{l}\right\}$, so that
  (30) $$\tilde{p}(\mu_{l})\propto p(\mu_{l})\exp\left(E_{q(w_{l},c_{l},\alpha_{l})}\ln\left\{p(w_{l}|\alpha_{l},c_{l},\mu_{l})\right\}\right)$$

For simplicity, the prior of the mean vector $\mu_{l}$ is set to be flat, and a delta VPD $q(\mu_{l})=\delta (\mu_{l}-\;{\hat{\mu }}_{l})$ is also applied. By maximizing $\tilde{p}(\mu_{l})$, a point estimation can be obtained as follows:

(31) $${\hat{\mu }}_{lk}^{\prime }=\frac{\sum_{n=1}^{N}{\hat{r}}_{lnk}{\hat{w}}_{ln}}{\sum_{n=1}^{N}{\hat{r}}_{lnk}}$$

### 3.3. Initialization Algorithm

The initialization algorithm is proposed to infer the initial variational variables, including the preliminary estimation of the parameter set Ω and the GMM model parameters. As shown in Algorithm 1, the estimation for multipath parameters $w_{l}$ and $\theta_{l}$ is obtained by cyclically finding and removing the corresponding template function $s(\theta_{l})$ from the remaining observations. The estimation process stops when the number of multipath reaches the preset amount $L_{0}$. This method will inevitably result in duplicated estimations with the same scatter parameter vectors. Therefore, the channel taps with the same $\theta_{l}$ are checked and combined to be one. After obtaining the multipath coefficients $w_{l}$, we use the expectation-maximization (EM) algorithm [21] to estimate the initial parameters of K Gaussian clusters. In the E-step, the probability that $w_{ln}$ is classified as the k-th Gaussian component, i.e., $p(c_{lnk}=1|w_{ln})$, is computed by the relative weight of the Gaussian likelihoods. In the M-step, $\mu_{lk}$ and $\Lambda_{lk}$ are updated by the statistics of observations. When the EM process converges, we obtain $r_{lnk}=p(c_{lnk}=1|w_{ln})$. Notice that when initializing the EM algorithm, improper mean and variance values may cause the estimation of $p(c_{lnk}=1|w_{ln})$ failing to converge. Thus in this paper, the K-means algorithm [26] is applied to estimate the initial mean and variance of each cluster.
        **Algorithm 1:** Initialization$\;\;\;\;\;\;\;\;\mathrm{Set}L\;=\;1,\;\alpha_{l}\;=\;0,\;r_{nk}=0,\;I\;=Ø;x_{l}\leftarrow z\;\;\;\;\;\;\;\;\;\mathrm{for}\;l\;=\;1\to L_{0}\;\;\;\;\;\;\;\;\;\;\;\;\;\;\mathrm{Initialize}\;q(\theta_{l})\;\mathrm{from}\;(25)\;\;\;\;\;\;\;\;\;\;\;\;\;\;\mathrm{Initialize}\;q(w_{l})\;\mathrm{from}\;(23)\;\;\;\;\;\;\;\;\;\;\;\;\;\;\mathrm{Initialize}\;q(\alpha_{l})\;\mathrm{from}\;(26)\;\mathrm{and}\;(27)\;\;\;\;\;\;\;\;\;\;\;\;\;\;{\hat{x}}_{ln}\leftarrow y_{n}-\sum_{l=1}^{L}{\hat{w}}_{ln}s({\hat{\theta }}_{l})\;\;\;\;\;\;\;\;\;\;\;\;\;\;I=I\cup l\;\;\;\;\;\;\;\;\;\;\;\;\;\;L=L+1\;\;\;\;\;\;\;\;\;\mathrm{end}\;\mathrm{for}\;\;\;\;\;\;\;\;\;\mathrm{//remove}\;\mathrm{duplicate}\;\mathrm{estimates}\;\;\;\;\;\;\;\;\;\mathrm{for}\;l\in I\;\;\;\;\;\;\;\;\;\;\;\;\;\;\mathrm{if}\;\forall d,\theta_{d}=\theta_{l}\;\;\;\;\;\;\;\;\;\;\;\;\;\;\;\;\;w_{l}=w_{l}+w_{d}\;\;\;\;\;\;\;\;\;\;\;\;\;\;\;\;\;I=I-d\;\;\;\;\;\;\;\;\;\;\;\;\;\;\;L=L-1\;\;\;\;\;\;\;\;\;\;\;\;\;\;\mathrm{end}\;\mathrm{if}\;\;\;\;\;\;\;\;\;\mathrm{end}\;\;\;\;\;\;\;\;\;\;\;\mathrm{//initialize GMM parameters}\;\;\;\;\;\;\;\;\;\mathrm{for}\;l\in I\;\;\;\;\;\;\;\;\;\;\;\;\;\;\mathrm{initialize}\;\mu_{l}^{0},\;\Lambda_{l}^{0}\;\mathrm{from}\;w_{l}\;\mathrm{by}\;\mathrm{K-means}\;\mathrm{algorithm}\;\;\;\;\;\;\;\;\;\;\;\;\;\;\mathrm{estimate}\;\mu_{l},\;\Lambda_{l},\;p(c_{lnk}=1|w_{ln})\;\mathrm{by}\;\mathrm{EM}\;\mathrm{algorithm}\;\;\;\;\;\;\;\;\;\;\;\;\;\;r_{lnk}=p(c_{lnk}=1|w_{ln})\;\;\;\;\;\;\;\;\;\mathrm{end}$

### 3.4. Pruning and Convergence Condition

A close examination of the sparsity prior (10) for the l-*th* multipath reveals that the sparsity parameter $\alpha_{lk}$ controls the width of the Gaussian prior. When $\alpha_{lk}\to \infty$ the cluster will be concentrated around the center which tends to be zero for a fading channel. Thus, large values of $\alpha_{l}$ will make the component “irrelevant” and be removed from the estimation. In the proposed method, we set $\sum_{k=1}^{K}\alpha_{lk}>TH_{p}$ as a pruning condition where $TH_{p}$ is a preset threshold. In order to halt the iterative variational process, another preset threshold $\Delta_{0}$ is used to check whether the estimation converges. Define the update rate for the *i*-*th* iteration

(32) $$\Delta^{\left(i\right)}=\frac{\sum_{l=1}^{L}\sum_{n=1}^{N}\left|w_{ln}{}^{\left(i\right)}-w_{ln}{}^{\left(i-1\right)}\right|}{\sum_{l=1}^{L}\sum_{n=1}^{N}\left|w_{ln}{}^{\left(i\right)}\right|}$$

**Figure 2:**
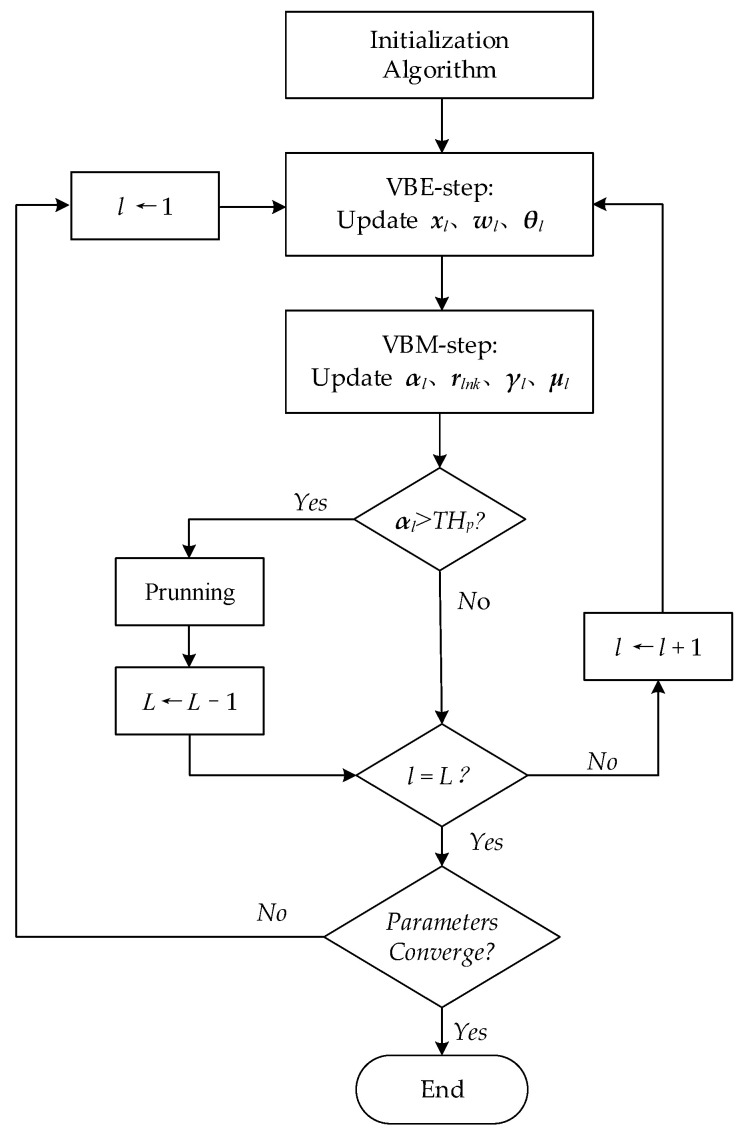
The flow chart of the VB-GMM algorithm.

The iteration process stops when $\Delta^{\left(i\right)}\leq \Delta_{0}$. The general flow chart of the VB-GMM algorithm is shown in Figure 2.

### 3.5. Computational Complexity

The computational complexity of the proposed algorithm is discussed in this section. For the benchmark comparison, we introduce the Gaussian prior-based variational Bayesian estimation scheme proposed in [5] (VB-G). The complex multiplication times (CMT) is used as the comparison metric for the evaluation of the computational complexity in VB-G and VB-GMM. Note that the multiplication of a L×M complex matrix and a M×N complex matrix demands the CMT of M×L×N. And the CMT required by computing the inversion of a N×N complex matrix is $4N^{3}$ [27]. The computational complexity of each procedure is given in Table 1, in which $T_{1}\text{\textasciitilde{}}T_{4}$ represents the number of iterations taken by the variational updates in VB-G, the EM process, the K-means algorithm, and the variational updates of the VB-GMM, respectively. Compared with VB-G, the initialization process of VB-GMM has greater complexity due to an iterative estimation for GMM parameters, in which the computational complexity of the K-means algorithm is $O\left(T_{3}NK\right)$ [28]. In the variational inference procedure, the coefficient of the main complexity term $ND^{3}$ in VB-GMM is much smaller, resulting in the running time of about 1/5 of the VB-G in numerical simulations.

## 4. Simulation Results

In the numerical simulation, the setup of the sounding parameters is given below. We use the constant amplitude zero auto-correlation sequence as the sounding sequence with 256 chips, and the interval is $T_{c}=5\;\mu s$. The sample rate at the receiver is set as $R_{s}=1\;\mathrm{MHz}$ which implies that the number of discrete samples per CIR is D=1280. The number of observations per WSS region is assumed as N=500. For the multipath channel, a 4-tap model is adopted in which the mean power of each tap is set as 0, −3, −5, −10 dB, and the multipath delay equals 10, 12, 15, 20 μs, respectively. For testing the performance of VB-GMM in various complex environments, three different fading types are considered. In the first scenario, the coefficients of the MPC are generated from Gaussian mixture distributions with K=3. The real and imaginary parts of Gaussian means, covariances, and the mixing coefficients are randomly selected from the interval [−10,10],[0,1] and [0,1], respectively. In the second scenario, all four multipaths are modeled as Weibull distribution with the shape parameter set as 3, 4, 5, 6, respectively. In the third scenario, we define the channel taps to be Nakagami-m distributed, and the m-parameters are set as 2, 3, 4, 5, respectively, to simulate different fading depths.

The numerical setup for the VB-GMM algorithm is as follows. The preset multipath amount is $L_{0}=20$, and the pruning threshold is set as $TH_{p}=10^{6}$ for all SNR conditions. In the VBE-step, the initial parameters are: $\alpha_{l}=0$, β=1 and a flat prior probability $p\left(\theta_{l}\right)$. In the VBM-step, the initial values of the parameters are: $a_{lk}=b_{lk}=10^{-7}$, $\gamma_{lk}=1/K$. Note that all the numerical simulations are based on over 5000 observations, and the iteration process is terminated when the update rate $\Delta^{\left(i\right)}$ is smaller than 0.001.

**Figure 3:**
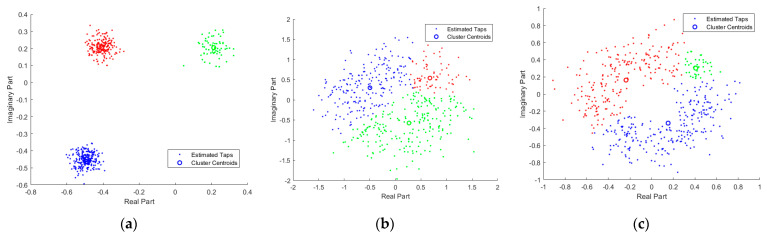
Scatter plot of estimated channel coefficients in different scenarios. (**a**) Gaussian mixture distributed channels; (**b**) Weibull distributed channels; (**c**) Nakagami distributed channels.

Figure 3a shows a scatter plot of the estimated Gaussian mixture channel taps with the noise level SNR=−2 dB, in which the circles represent cluster centroids and the dots represent the estimated channel taps. The graph indicates that when the channel coefficients are Gaussian mixture distributed, the proposed VB-GMM is capable of classifying channel tap samples that come from different Gaussian components. Meanwhile, the cluster centroids can be precisely located. In Figure 3b,c, we examined the classification capability of the proposed algorithm when the channel taps are assumed to be Weibull and Nakagami distributed, respectively. As shown in Figure 3b,c, the channel coefficients in Weibull and Nakagami scenarios with random phases are clearly clustered into three regions, which indicates that the proposed method can learn and fit the distribution of random channels.

**Figure 4:**
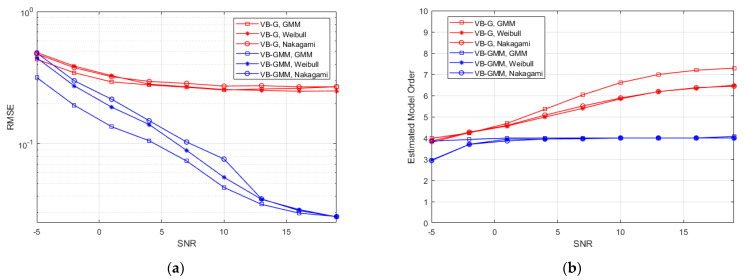
(**a**) RMSE between the synthetic and reconstructed channel responses; (**b**) The mean value of estimated model order.

**Figure 5:**
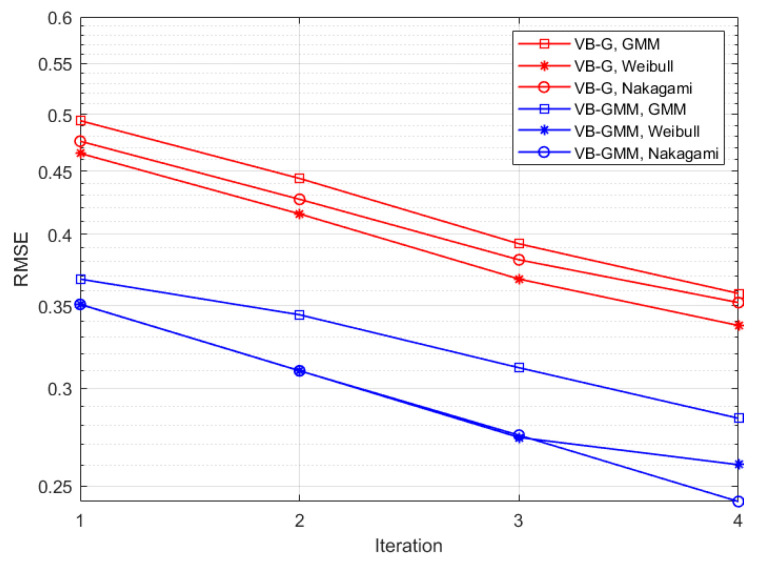
The RMSE of estimated channel responses versus iteration numbers.

Figure 4a represents the RMSE between the synthetic and reconstructed CIRs versus SNR in different scenarios. Notice that the VB-G scheme adopts a fixed sensitivity level of SNR′=10 dB. For low SNR of −5 dB, the VB-GMM exhibits comparable performance with the VB-G. As SNR increases, the VB-GMM clearly outperforms the VB-G in all three scenarios due to the flexibility in fitting non-Gaussian data. The reason is that for VB-SAGE the estimation errors of $\theta_{l}$, i.e., the delay of MPCs in part of the observations will result in a significant RMSE between the synthetic and reconstructed channel responses. While for VB-GMM, the multipath observations make the estimate of $\theta_{l}$ more robust because $\theta_{l}$ is assumed invariant in the WSS region. Figure 4b shows the curves of the estimated mean model order versus SNR. In the SNR range of −5 dB to 4 dB, the value of sparsity parameters tends to diverge, resulting in an obvious suppression effect on the MPCs. Under most SNR conditions, the VB-GMM shows accurate model selection for the 4-tap multipath channel, while the VB-G has a positive model order bias. Because for some of the observations if $\theta_{l}$ is wrongly estimated, there will be residual in the received signal after removing the template function, which leads to the falsely identified components. From the results of Figure 3 and Figure 4, the proposed method demonstrates its superiority in terms of model complexity and estimation accuracy. In Figure 5 we demonstrate curves of RMSEs that vary with the increasing iteration number in different scenarios for an SNR of 10 dB. The proposed method performs a similar convergence rate to the Gaussian prior-based scheme with smaller RMSEs in all the scenarios.

**Figure 6:**
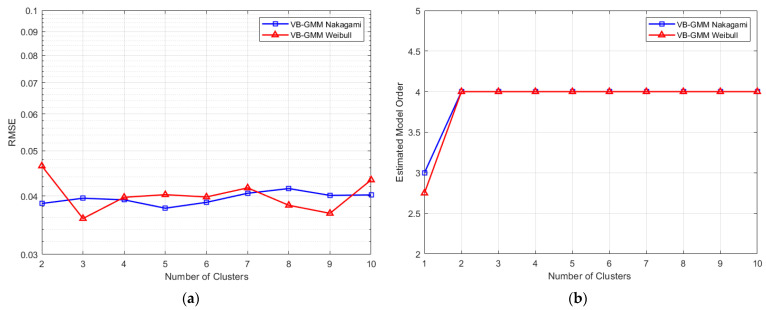
The performance of VB-GMM versus the number of Gaussian components. (**a**) RMSE between the synthetic and reconstructed channel responses; (**b**) The mean value of estimated model order.

To study the influence of the number of Gaussian components K, we generated over 30,000 channel responses from the Weibull and Nakagami channels and the performance of the proposed VB-GMM is given in Figure 6. In the simulation, the SNR is fixed at 10 dB. As depicted in Figure 6a, the RMSEs of the reconstructed channel responses have no significant change as K increases in both Weibull and Nakagami channels. From Figure 6b, it can be seen that the estimated model orders are accurate when K≥2. Consider that a large K cannot bring considerate improvement and may increase the complexity of GMM, we propose to set K=3 for most channel conditions.

## 5. Conclusions

In this paper, we presented a variational Bayesian-based channel parameter estimation approach for Gaussian mixture distributed wireless channels, which combined the variational Bayesian estimation [5] and the variational GMM training [21]. Compared with the existing Bayesian estimation schemes, the proposed scheme can exploit the sparse solutions of the multipath channel and represent the complicated distribution patterns by the application of GMMs. Experiment results showed that the VB-GMM method can obtain the channel estimates with a small reconstruction RMSE and determine the optimal number of MPCs through a fixed pruning criterion under various SNR conditions. For future works, we will further investigate factors that may affect the algorithm performance in terms of the sparsity and the implementation complexity.

## Figures and Tables

**Table 1:** The CMT required by VB-G and VB-GMM algorithms.

Procedure	VB-G	VB-GMM
Initialization	$\left(6D^{3}+4D^{2}+\frac{1}{2}DL_{0}\right)NL_{0}$	$\left(ND{\left(D+1\right)}^{2}+4N^{2}K+\frac{1}{2}DL_{0}\right)L_{0}+\left(D^{2}+K\right)NL_{0}T_{2}+O\left(T_{3}NK\right)$
Variational inference	$\sum_{t=1}^{T_{1}}NDL_{t}+\left(6D^{3}+3D^{2}+3D\right)NT_{1}$	$\sum_{t=1}^{T_{4}}NDL_{t}+\left(ND{\left(D+1\right)}^{2}+N\left(K^{2}+2K+4\right)+\left(4N^{2}+2\right)K\right)T_{4}$

## References

[1] Fleury B.H., Tschudin M., Heddergott R., Dahlhaus D., Pedersen K.I. (1999). Channel parameter estimation in mobile radio envi-ronments using the SAGE algorithm. IEEE J. Sel. Areas Commun..

[2] Liu T.C.-K., Kim D.I., Vaughan R.G. (2007). A High-Resolution, Multi-Template Deconvolution Algorithm for Time-Domain UWB Channel Characterization. Can. J. Electr. Comput. Eng..

[3] Lanterman A.D. (2000). Schwarz, Wallace, and Rissanen: Intertwining themes in theories of model order estimation. Int. Stat. Rev..

[4] Myung J.I., Navarro D.J., Pitt M.A. (2006). Model selection by normalized maximum likelihood. J. Math. Psychol..

[5] Shutin D., Fleury B.H. (2011). Sparse Variational Bayesian SAGE Algorithm with Application to the Estimation of Multipath Wire-less Channels. IEEE Trans. Signal Process..

[6] Bajwa W.U., Haupt J., Sayeed A.M., Nowak R. (2010). Compressed Channel Sensing: A New Approach to Estimating Sparse Multipath Channels. Proc. IEEE.

[7] Tipping M.E. (2001). Sparse bayesian learning and the relevance vector machine. J. Mach. Learn. Res..

[8] Thomas B. (2013). Variational Sparse Bayesian Learning: Centralized and Distributed Processing. Ph.D. Thesis.

[9] Badiu M., Hansen T.L., Fleury B.H. (2017). Variational Bayesian Inference of Line Spectra. IEEE Trans. Signal Process..

[10] Yuan W., Wei Z., Yuan J., Ng D.W.K. (2020). A Simple Variational Bayes Detector for Orthogonal Time Frequency Space (OTFS) Modulation. IEEE Trans. Veh. Technol..

[11] Hu B., Land I., Rasmussen L.K., Piton R., Fleury B.H. (2008). A Divergence Minimization Approach to Joint Multiuser Decoding for Coded CDMA. IEEE J. Sel. Areas Commun..

[12] Zhong K., Wu Y., Li S. (2013). Signal Detection for OFDM-Based Virtual MIMO Systems under Unknown Doubly Selective Chan-nels, Multiple Interferences and Phase Noises. IEEE Trans. Wirel. Commun..

[13] Bai X., Zhang Y., Zhou F. (2018). High-Resolution Radar Imaging in Complex Environments Based on Bayesian Learning with Mixture Models. IEEE Trans. Geosci. Remote Sens..

[14] Karseras E., Dai W., Dai L., Wang Z. Fast variational Bayesian learning for channel estimation with prior statistical infor-mation. Proceedings of the 2015 IEEE 16th International Workshop on Signal Processing Advances in Wireless Communications (SPAWC).

[15] He R., Zhong Z., Ai B., Wang G., Ding J., Molisch A.F. (2012). Measurements and Analysis of Propagation Channels in High-Speed Railway Viaducts. IEEE Trans. Wirel. Commun..

[16] Matolak D.W., Sen I., Xiong W. (2008). The 5-GHz Airport Surface Area Channel—Part I: Measurement and Modeling Results for Large Airports. IEEE Trans. Veh. Technol..

[17] Sen I., Matolak D.W. (2008). The 5-GHz Airport Surface Area Channel—Part II: Measurement and Modeling Results for Small Airports. IEEE Trans. Veh. Technol..

[18] Yu J., Chen W., Li F., Li C., Yang K., Liu Y., Chang F. (2020). Channel Measurement and Modeling of the Small-Scale Fading Characteristics for Urban Inland River Environment. IEEE Trans. Wirel. Commun..

[19] Tan S., Huang K., Shang B. (2017). Sparse Bayesian Learning with joint noise robustness and signal sparsity. IET Signal Process..

[20] Pedersen N.L., Manchon C.N., Shutin D., Fleury B.H. (2012). Application of Bayesian Hierarchical Prior Modeling to Sparse Channel Estimation. IEEE Int. Conf. Commun..

[21] Tzikas D.G., Likas A.C., Galatsanos N.P. (2008). The variational approximation for Bayesian inference. IEEE Signal Process. Mag..

[22] Lee W. (1985). Estimate of local average power of a mobile radio signal. IEEE Trans. Veh. Technol..

[23] Fessler J.A., Hero A.O. (1994). Space-alternating generalized expectation-maximization algorithm. IEEE Trans. Signal Process..

[24] Babacan S.D., Molina R., Katsaggelos A.K. (2009). Bayesian Compressive Sensing Using Laplace Priors. IEEE Trans. Image Process..

[25] Bishop C.M. (2006). Pattern Recognition and Machine Learning (Information Science and Statistics).

[26] McQueen J.B. (1967). Some methods of classification and analysis in multivariate observations. Proceedings of the Fifth Barkley Symposium on Mathematical Statistics and Probability.

[27] Kusume K., Joham M., Utschick W. MMSE block decision-feedback equalizer for spatial multiplexing with reduced com-plexity. Proceedings of the IEEE Global Telecommunications Conference 2004.

[28] Pakhira M.K. A Linear Time-Complexity k-Means Algorithm Using Cluster Shifting. Proceedings of the 2014 International Conference on Computational Intelligence and Communication Networks.

